# 

**Published:** 1881-04

**Authors:** 


### INDEX.

### VOLUME XVII. TO XIX. INCLUSIVE.

**Contributions.**

PAGE.
A Neglected Part of Education, J. J. Keyes .......... 34
American Architecture, Ausburn Towner .......... 238
A Political Picnic, T. K. Beecher, H. H. Rockwell, C. A. Collin, and E. W. Krackowizer .......... 84
Architecture. Ausburn Towner .......... 211
Bacteria, S. O. Gleason .......... 60
Blind Man's Buff, George E. Blackham, M. D. .......... 138
Capital Impressions. Ben H. Pratt .......... 1
Cheek—Does it Pay? Ben H. Pratt .......... 53
Combinations, J. B. Chandler .......... 136
Communicable Diseases, George E. Blackham, M. D. .......... 163
Concerning the Apprentice System, Ben. H. Pratt .......... 183
Culture, J. B. Chandler .......... 296
Diphtheria and Scarlet Fever, Exchange .......... 61
Doctors, Ancient and Modern, Ausburn Towner, I. .......... 55
Doctors, Ancient and Modern, Ausburn Towner, II. .......... 82
Doctors, Ancient and Modern, Ausburn Towner. III .......... 108
Doctors Born Not Made, Ausburn Towner .......... 134
Domestic Remedy for Felons, Dr. T. C. Brannon .......... 9
Give Us a Rest, Geo. E Blackham, M. D .......... 266
(And Reply by E. J. Dunning.)
Hats and Baldness, Ben. H. Pratt .......... 209
Heredity. Jas. B. Chandler .......... 57
How Do Men Become Doctors? Ben. H. Pratt .......... 287
Infinitessimalism versus Science, C. S Carr, M. D .......... 245
Infinitessimalism versus Science, H. M. Corey, M. D .......... 269
(And Reply by Dr Carr.)
Infinitessimalism Again, C. S. Carr, M. D .......... 292
Invisible Blood Corpuscles, J. M. Adams .......... 268
Laughter, Ausburn Towner .......... 186
Music, Ausburn Towner .......... 159
Myths, Ausburn Towner .......... 263
Names, Ausburn Towner .......... 290
Our Cantata. Ausburn Towner .......... 29
Parents and Children. Their True Relation, Ben. H. Pratt .......... 235
Penalties and Crimes, W. E. Knox, Thomas K. Beecher and J. B. Chandler .......... ...
PAGE.
Preliminary Education for Medical Students, George E. Blackham, M. D. ..........
Protoplasm, S. O. Gleason, M. D. ....
Rambling. J. B Chandler .......... 268
Retrospection. J. B. Chandler .......... 191
Secret of the Servant Girl Trouble, Ben. H. Pratt .......... 131
Shall I Send My Boy to College? Ben. H. Pratt .......... 261
Specialties, James B Chandler .......... 215
State Education, Theophrastus Schopedhaver, I. .......... 59
State Education, Theophrastus Schopenhaver, II. .......... 140
Suggestions, Educational, Ben. H. Pratt .......... 27
The Book Wonderful, Ausburn Towner .......... 4
The Care of the Eyes, Geo. E. Blackham, M. D .......... 243
The Late Architectural Craze, Ben. H. Pratt .......... 157
The War Cure, J. B. Chandler .......... 36
Thoughts on General Education, J. B. Chandler .......... 242
To Prevent the Spreading of Scarlet Fever, J. Davidson, M. D. .......... 8
Trouble. J. B. Chandler .......... 165
Trouble. J. B. Chandler .......... 91
What of It? Geo. E. Blackham, M D. 294
Wherewith I Shall We Be Clothed? Ben. H Pratt .......... 105
Words and Slang, J. B Chandler .......... 7
Worried to Death, Ben. H. Pratt .......... 79

**Medical Items and News.**

Abdominal Typhus .......... 12
Abortion .......... 198
Absorbent Powders .......... 194
Acne .......... 247
Acute Bronchitis .......... 65
Acute Rheumatism .......... 41
After Pains .......... 217
Ague .......... 167
Albuminuria .......... 300
Albuminous Obstruction of the Bladder .......... 38
Albuminuric Retenitis of Pregnancy. 252
Alcoholism .......... 93
Alimentation, Rectal .......... 118
Alkaline Draughts .......... 167
Alopæcia and Seborrhœa .......... 169
Amenorrhœa .......... 122
A Movement Against Vivisection .......... 217
Amyl Nitrate as a Remedy for Ague. 195
Anæsthetics, Local .......... 279
Anæsthetic Mixture at St. Bartholomew's Hospital .......... 146
PAGE.
Angina Faucium .......... 301
Angina Pectoris .......... 223
Anti-Asthmatic Mixture .......... 194
Anti-hemorrhagic Boli .......... 272
Antipruritic .......... 168
Antipyretic .......... 195
Antiseptic, Catgut .......... 38
Antiseptic, Quinia .......... 117
Antisuppurative .......... 173
Aperient, Simple .......... 147
Argyria .......... 13
Asclepias Curassavica .......... 250
Asthma ..15–37–64–66–96–170–194–198–273
Asthmatic Paroxysm .......... 64
Atropia in Choraee .......... 118
Aural Vertigo .......... 172
Bacillus Malarial .......... 280
Bacillus of Syphilis, Cultivating .......... 196
Backache, Its Cause .......... ...
Basham's Mixture .......... 247
Barber's Itch .......... 12
Baths for the Newly Born .......... 147
Battey, Solution for Eczema .......... 11
Beef Tea .......... 197
Bee Stings .......... 16
Biliary Calculi .......... 67
Bilious Colic .......... 63
Bismuth, Death from Undigested .......... 170
Bladder, Albuminous Obstruction of 38
Bleaching Sponges .......... 271
Blennorrhagia .......... 148
Blood, Microscopic Examination of. 13
Boils .......... 276
Boracic Acid as a Surgical Dressing. 248
Bougies, Gelatine .......... 37
Bright's Disease .......... 123–169
Bronchial Affections of Pulmonary Phthisis .......... 120
Bronchitis, Acute .......... 65–218
Bronchitis, Chronic .......... 17–192–194
Bubces .......... 276
Burns .......... 10–38–68–92–93–272
Burns from Sulphuric Acid .......... 38
Calcium Sulphide in Small pox .......... 169
Calculi, Biliary .......... 67
Calomel in Small Doses .......... 93
Camphor, Compound Powder of .......... 16
Cancer .......... 299–250
Cancer of Uterus .......... 220
Carbolized Iodoform .......... 249
Carbonated Laxative Water .......... 38
Castor Oil .......... 13
Castor Oil, New Mode of Administering .......... 144
Catarrh .......... 62–145–171–273–274
Catarrhal Conditions .......... 223
Catarrh, Vesical .......... 168–219
Catgut Antiseptic .......... 38
Caustic Powder, Esmarck's .......... 10
Cellulose as a Dressing .......... 279

PAGE.
Cerumen, Impaction of............ 274
Chalky Deposits.................... 300
Cheken ................................ 14
Chest Affections...... ............... 14
Chilblains............................ 62
Chilblain Liniment................ 194
Chilblains, Remedy for............. 10
Chloral Hydrate and Diabetes...... 121
Chloral Hydrate as a Vesicant...... 251
Chlorosis, Anemia of............... 92
Cholera Infantum.........62-92-117-147
Cholera Mixture..............62-62-278
Chordee ............................. 247
Chorea.......................195-201-277
Cod Liver Oil, Tasteless........... 93
Colic.................................. 272
Colic, Bilious........................ 63
Colic, Habitual.... .................. 40
Colloid Styptic...................... 37
Collodion, Styptic................... 10
Collyrium ............................ 168
Condylomata......................... 300
Conjunctiva ......................... 218
Constipation ..65-97-149-194-199-222-249
Constipation in Infants............ 173
Contagious Diseases................ 248
Contagiousness of Tubercular Matter 118
Convalaria Majalis.................. 272
Convulsions, Infantile............92-146
Covering the Taste of Bromides..... 92
Coughs and Night Sweats of Phthisis. 62
Cough Mixtures.............200-217-221
Cough, Spasmodic.......... 62-276-217
Creosote Wine........................ 275
Croup ........................93-95-169
Croupous Pneumonia............... 63
Cystitis.... .....10-15-38-62-194-195-273
Delirium Tremens.................. 276
Delphinium Ajacis.................. 170
Diabetes .........38-146-168-247-275-280
Diarrhœa .......................62-147-169
Diarrhœa, Chronic.................. 247
Diarrhœa of Infants...... .. 14-220-221
Diethylacetate and Dimethylacetate as an Anæsthetic.................. 272
Diphtheria...10-17-37-66-68-97-121-123-124-171-196-198-219-220-249-249-276
Diuretic Tea......................... 273
Dropsy ................................ 11
Dysentery......................62-120-219
Dysmenorrhœa...................... 196
Dyspepsia........................195-274
Dyspepsia in Infants............... 97
Dyspnœa ............................. 301
Dyspnœa, Cardiac................... 17
Dyspnœa, Quebracho Bark in..... 150
Earache............................... 95
Ear, Abscess of the Lobule of the.... 301
Eclampsia.......................63-64-247
Eczema............. 92-168-200-221-277
cze m a, Battey Solution for........ 11
Eczema, Chronic.................... 200
Eczema Genitalia.................174-222
Elixir, Simple....................... 250
Embalming .......................... 174
Emmenagogue ......119-147-171-250-276
Enema, Quinine and a Solution of Beef ................................. 198
Ephelis ............................... 301
Epilepsy ................119-151-202-219
Epilepsy, Bromides in.............. 94
Epistaxis ............................. 251
Epithelioma of the Neck of the Uterus ................................... 120
Ergot in Obstetrics................. 197
Ergot, Therapeutical Indications for 144
Ergotin, Danger of.................. 92
Eructations, Acid................... 195
Erysipelas........63-147-167-220-273-299
Erysipelas, Facial........63-122-169-248
Esmarck's Caustic Powder......... 10
Eye Lotions.......................... 278
ye Lotions, Distilled Water for... 278
Facial Erysipelas..................39-63
Fatty Degeneration of the Heart... 168
Felons................................ 95
Fetid Bronchitis..................... 302
Fetid Foot Sweat.................... 275
Fever and Ague...................... 12
Fissured Nipples..................94-280

PAGE.
For Imperfection in Speech........ 299
Freckles ......................38-63-300
Gall Bladder, Excisiou of the....... 302
Gastralgia ........................... 299
Gastric Flatulence, Acidity and Pyrosis ................................. 39
Gastric Ulcer........................ 299
Gauze, Lister's Eucalyptus......... 119
Gelatine Bougies.................... 37
Gleet ................................. 11
Glycerine Tonic...................... 11
Glycerite of Starch.................. 277
Gonorrhœa .....................12-92-119
Gonorrhœal Ophthalmia............ 93
Gonorrhœa Suppositories........... 170
Gout...............................66-248
Gout, Sub-Acute..................... 39
Growth of Hair...................... 64
Guachamca ......................... 272
Hæmoptysis...................... 277-301
Hæmorrhages, Uterine.............. 119
Hæmorrhoids ..............117-168-249
Hæmorrhoids, Bleeding............ 195
Hair, Growth of..................... 64
Hair Restorer........................ 247
Hay Fever, Treatment of........... 93
Headache.......118-121-198-217-224-250
Heart Disease................. 16-247-274
Heart's Action, Restoring of...... 67
Herpes Zoster................12-167-247
Hiccough .......................145-280
Hoarseness........................... 198
Hydrastis Canadensis.............. 273
Hydrobromic Acid................... 222
Hydrocele....................174-199-277
Hyperidiosis and Urticaria......... 272
Hydrophobia Treated with Pilocarpine .................................. 149
Hypodermics in Syphilis........... 16
Hysteria ............................. 117
Hysteria and Epilepsy.............. 64
Incontinence of Urine............... 218
Incontinence of Urine, Nocturnal.. 195
Indian Hemp, Medicinal Use of..... 93
Infants, Diarrhœa in................ 14
Infant Food.......................... 248
Infectious Diseases.................. 300
Inhalation of Vapors................ 13
Insomnia ............................. 117
Insomnia and Asthma............... 96
Intermittents ........................ 195
Intermittent Fever, Chronic........ 98
Iodoform ............................. 37
Iodoform, Covering Odor of ....... 275
Iodoform, Solution for Hypodermic Use .................................. 277
Iron Lemonade....................... 199
Iron Pomade, Muriate Tincture of.. 147
Iron Preparations................... 146
Iron, Tincture of Ferri per Chloridi (pleasant preparation) ......... 167
Itch ...........................15-63-252
Itch, Barber's........................ 12
Jequirity ............................. 277
Kava Kava........................... 250
Kissing, Danger of.................. 300
Laryngeal Phthisis.................. 117
Leeches, To Keep................... 40
Leucorrhœa..............12-174-217-222
Lock-jaw ............................. 66
Lotion, Kummerfeld's............... 118
Lumbago ............................ 276
Lumbricoides........................ 301
Malaria .............................. 171
Malarial Hematuria.................. 299
Malarial Poisoning, Chronic........ 62
Mammæ, Inflammation of..........62-92
Menstrual Flow...................... 251
Menstrual Flow Scanty and the Liver Sluggish............................ 144
Mercurial Poisoning................. 301
Metallic Foreign Bodies in the Eye.. 196
Metritis, Acute....................... 169
Micrococci ........................... 223
Micro Organisms.................... 171
Micro Organism of Pneumonia..... 275
Microscopical Examination of Blood. 13
Migraine ............................. 276
Mitral Affections.................... 144
Morphia to the moribund.......... 117
Mixed Chloroform.................. 302

PAGE
Nævus, Danger of Injection of Iron in..................................... 14
Nephritis, Chlorate of Potassium in. 14
Neuralgia ..122-168-217-218-221-274—277
Neuralgia of the Stomach.......... 194
Night Sweats................117-118-248
Nipples, Sore...................146-167
Nitro-Glycerine ..................... 16
Nocturnal Incontinence of Children 302
Odor of the Lochia.................. 217
Olive Oil in Diseases of the Chest... 194
Opium Poisoning, Coffee Hypodermically.................................. 145
Orchitis.........................223-248
Otorrhœa, Dry Treatment ......... 274
Ovaritis.............................. 247
Perforation of Membrani Tympani from Ascaris Lumbricoides..... 42
Pharyngitis.......................... 16
Phthisis........................252-276
Phthisis, Purgative in.............. 14
Phthisis and Catarrhal Cough...... 194
Piles ...........................218-276
Piles, Blind.......................... 145
Pilocarpine .......................... 43
Pitting of Small-pox ............... 37
Pleuritic Pain in Pneumonia........ 247
Pneumonia ........................96-146
Pneumonia, Croupal................ 145
Podophyllin.......................... 94
Potassium, Chloride................. 196
Preserving Tissues and Embalming Bodies, A New Method of..... 148
Prolapsus Ani.....................40-65
Prurigo .............................. 144
Prurigo and Eczema................ 169
Pruritis.................15-167-174-222
Psoriasis........................144-217
Purgative for Consumptives......... 39
Pustule, Malignant.................. 218
Quebracho ........................... 63
Quinine, Disguising the Taste of.... 220
Quinine, Hypodermic Use of....... 199
Quinine, To Hasten the Action of.. 145
Rabies................................. 172
Rectal Alimentation................. 118
Relative Mortality Between White and Colored Races............... 167
Restoring the Heart's Action........ 67
Retinitis, Albuminurica............ 252
Rheumatism .....41-147-149-194-202-217-249-251
Rheumatism, Acute Infantile....... 273
Rheumatism, Chronic............64-121
Rheumatic Fever..................... 273
Rhus Aromatica...................... 65
Ringworm.......................39-218
Salicylic Acid, Menstrum for....... 13
Salicylic Acid in Small-pox......... 95
Salicylic Ointment.................. 217
Scabies .......................15-63-252
Scarlatina....................98-173-274
Scarlet Fever......................... 274
Scars .................................. 300
Scrofula and Tuberculosis.......... 151
Secondary Syphilis.................. 301
Sewer Gas Poisoning................ 302
Skin Affections...............68-96-250
Small-pox, Pitting.................16-37
Snake Bites....................247-248
Soda Mint.......................11-277
Sodium, Ethylate of................. 95
Sodium Phosphate in Habitual Colic 40
Soft Chancres and Buboes ........ 276
Sore Nipples......................... 64
Spasm of the Glottis................ 217
Spasmodic Cough................... 299
Spasms of the Œsophagus.......... 300
Spermatorrhœa ...................... 118
Squint Cured Without an Operation. 275
Stimulating Properties of Oats..... 271
Strychnia, Antidote for Alcohol.... 17
Stye.................................... 249
Styes, Abortive Treatment of....... 199
Styptic Collodion ................... 10
Styptic Colloid...................37-167
Sub-Acute Gout and Rheumatism.. 39
Sub-Acute Pleurisy.................. 65
Submammary Intercostal Neuralgia. 274
Summer Dysentery of Children..... 62
Summer Diarrhœa of Children...... 65

PAGE.
Sweating....................196–221–273
Sweating of Hands and Feet........ 280
Sweating of Phthisis............276–279
Sycosis ............................ 198
Syphilis......... ........10–117–147–300
Syphilis and Vaccinia, Antagonism Between.......................... 299
Syphilis, Hypodermic Formula for. 16
Syphilitic Neuralgia ............... 10
Tænia .....................41–118–252
Tape Worm..................41–118–252
Teething Infants.................. 277
Temperature, High................. 144
Tetanus............................ 66
Tic Douloureux..................... 40
Tincture Ferri Perchloridi......... 197
Tin Poisoning...................... 299
To Keep Leeches.................... 40
Tonic, Alterative............ .....249–275
Tonga ............................. 14
Tonic Glycerine.................... 11
Tonic, Loomis...................... 219
Tonsilitis .............. 124–170–219–299
Toothache ......................... 38
Toothache, Neuralgic............... 279
Transfusion ....................... 122
Trichinosis Nodules................ 300
Turpentine, Oil of, in Mixtures.... 145
Tuberculosis and Scrofula.......... 151
Typhoid Fever...................... 192
Typhoid and Intoxication........... 299
Typhoid Fever, Coffee in........... 248
Typhoid State...................... 37
Typhus and Typhoid................. 302
Ulcers ............................ 197
Ulcers in Ear...................... 217
Ulcer of the Cornea, Scrofulous.... 201
Urinary Test Pellets, Formula for.. 280
Ulcers, Tongue..................... 272
Urethritis......................... 11
Urine, Alkalies in the............. 172
Urine, Disinfection of............. 123
Urine, Hydrate of Chloral for Preserving .... .................. 146
Urine, Retention of, Cockle Burr in. 196
Urticaria ......................... 272
Urticaria Produced by the Use of Tincture Pimpinallæ........... 39
Uterine Complaints, Tonic in....... 196
Uterine Engorgement................ 11
Uterine Sedative................... 37
Vaccine Virus...................... 43
Vaginal Catarrh.................... 12
Vaginitis.......................... 12
Vapors for Inhalation.............. 13
Varicose Veins..................... 247
Vermifuge..........................15–276
Vivisection, Movement Against...... 217
Vomiting .......................... 221
Vomiting, Obstinate................ 197
Vomiting in Pregnancy.............. 250
Warts, To Remove .................. 242
Warts, Venereal and Common......... 221
Weak Heart—Electricity vs. Chloroform............................ 301
Whooping Cough.........17–121–219–300
Winter Eczema...................... 299
Worms, Intestinal.................. 120
Wounds, Antiseptic Powder for.... 147
Zoster, Oil of Peppermint in........ 299

**Correspondence.**

Letters with Answers..... 18–41–68–142

**Household Hints and Recipes.**

A Bronze ......... : ............... 303
Adhesive Liquid for Metal.......... 226
A Disinfectant Laundry Blue........ 303
Anneal Lamp Chimneys............... 45
Bee Stings, Salicylic Acid for..... 45
Black Lacquer for Coating Bottles.. 303
Black Walnut, To Imitate........... 175
Bleaching Vegetable Tissues........ 226
Carpet Beetle...................... 20
Cement for Amber................... 203
Cement for Aquaria or Water Tanks 253
Cement for Fire Proof.............. 70
Cement for Glass................... 175
Cement for Glass and Metals........ 176
Cement for Leather................. 19
Cement for Mica.................... 175
Cement for Rubber.................. 45
Chilblains......................... 203
Coating Insects, Leaves and Flowers with Copper......... .......... 203
Coffee and Egg for Sick Persons.... 71
Cold Cream......................... 226
Color, Iron Black.................. 125
Copies of Drawings................. 253
Corns..........................175–225
Cream Mead......................... 246
Dangers of Mouldy Bread............ 70
Diamond Ink........................ 20
Diamonds, To Distinguish........... 20
Drains, Smoke Test for............. 281
Drinks for Fever Patients.......... 46
Dye for Feathers .................. 125
Dyeing Billiard Balls.............. 125
Dyeing Orange Yellow, Onion Peel.. 225
Embalming Bodies and Preserving Tissues, New Method............ 125
Emery, Ashes a Substitute for ..... 45
Fat Stains on Paper, To Remove ... 252
Felons............................. 225
Fire and Water Proof Paper......... 175
Florida Water...................... 203
Freckles, To Remove................ 176
Furniture Polish................... 19
Germination of Seeds, Rapid........ 70
Glass, To Drill.................... 20
Glucose ........................... 46
Glue, Elastic...................... 19
Glue, To Resist Fire .............. 226
Glue, Liquid...................45–71–253
Glycerine for the Hair............. 225
Grape Leaves for Pickles........... 303
Grease Spots, Removal of........... 99
Ground Glass, Imitation of......... 99
Harness Soap....................... 226
Imitation Walnut................... 303
Incombustible Curtains............. 225
Ink, Diamond....................... 20
Ink for Marking.................... 253
Ink for Rubber Stamps........ 226–253
Ink, Red........................... 175
Iron, To Preserve from Rust....... 17ç
Ink Spots, To Remove............... 70
Kumys ............................. 253
Label Varnish...................... 303
Lacquer, Brass..................... 254
Leather, To Render Water-proof... 71
Marble or Alabaster, To Clean...... 99
Moths, To Destroy.................. 226
Nickel Plating..................... 304
Oak Framing, To Darken............. 45
Oil of Eggs........................ 226
Oxidizing Silver................... 70
Paint, Composition for Removing.. 226
Paint or Varnish for Blackboard.... 254
Paint, To Clean.................... 225
Paint, Transparent, for Glass...... 19
Paste for Mounting Photographs... 176
Pencil Marks, To Fix............... 19
Pens, How to Clean................. 203
Perfume for Violet Powder.......... 203
Potato Salad....................... 19
Posts, Wood, To Preserve........... 99
Preserve Cider, Salicylic Acid...... 176
Preservation of Fruit.............. 303
Preserve Starch Paste.............. 70
Prize Receipt for Salad............ 304
Profilograph, The.................. 99
Removal of Encaustic Letters....... 281
Renovating Old Oil Paintings...... 281
Restoring the Finish of Leather.... 303
Roaches, To Destroy................ 176
Rust, To Remove.................... 19
Shaving Soap....................... 304
Shoe Dressing, Ladies'............. 70
Shoe Varnish....................... 203
Silver Oxidizing................... 70
Silvering Non-Metallic Substances.. 45
Slate, Preparation of Artificial...... 176
Smoke Test for Drains.............. 281
Soot Stains on Marble ............. 253
Spruce Beer........................ 99
Stain for Mahogany Cherry ... 175
Starch Gloss, Liquid .............. 71
Substitute for Stamping Pad........ 281
Sweating Hands or Feet, Remedy for 226
Teeth, Preservation of............. 20
Thomas's Electric Oil.............. 303
To Clean Brass..................... 304
To Embalm Small Quadrupeds......... 304
To Keep Wooden Posts from Rot... 303
Toothache Remedy................... 203
Toothache, Treatment of............ 71
Tooth Powder, Charcoal............. 70
To Transfer Figures, Names, Pictures, etc., Upon Glass......... 45
Transparent Paint for Glass........ 19
Varnish, Aqueous Shellac........... 175
Varnish for Preventing Rust........ 70
Waterproof Canvas for Covering Carts............................ 125
Worcestershire Sauce............... 46
Writing Fluid, Red................. 175

**Editorial.**

A Bad Element...................... 180
A Beautiful Simile ................ 73
Abdominal Surgery.................. 204
Advantages and Disadvantages. 206
Advertising Quackery............... 230
A Kick ...... ..................... 152
Always a Little Sick............... 205
A Magnificent Cabinet.............. 231
Amateur Photography................ 152
Amended Statement.................. 231
"A Merry Christmas."............... 305
American Society of Microscopists 74–152–180–234
A Morgan Monument.................. 180
An Arrival......................... 232
Ancestral ......................... 47
A New Motto........................ 126
Another Death ..................... 283
Another New Contributor............ 255
An Unusual Funeral...... ......... 228
A Projecting Microscope............ 228
A Silver Medal..................... 73
A Small Subscription............... 305
Athletes, The...................... 126
Autumn Days........................ 177
Bacteria and Dr. Gregg............. 282
Banqueting a Microscopist.......... 230
Bart's Experiment.................. 256
Baxter and Bliss, Drs.............. 49
Baxter, Dr. J. H................... 23
Beecher, C. M...................... 48
"Before the Class."................ 306
Big Bass........................... 47
Bishop and the Squirrel............ 73
Blood Corpuscles................... 206
Bodines............................ 22
Body Snatching..................... 204
Books and Publications......104–182–208
Bridge, Over Railroad Piers........ 23
Buying Up Manufacturing Concerns 229
Canadian Residence................. 100
Catastrophe ....................... 25
Change in Editorial Headings...... 22
Checks on Dishonesty............... 284
Chrismas Again..................... 205
Child Murder....................... 204
Clean Up .......................... 24
Cooling a Watermelon............... 73
Coroners' Inquests................. 24
Cremation Again.................... 284
Cremation Society.................. 23
Crowded Out ....................... 126
Danger of Inhumiaton............... 305
Deserved Appointment............... 255
"Digger Indian Rugs."............. 255
Doctors Blackham and Dunning on Carlyle............................ 282
Doctor Bliss and Doctor Hammond. 73
Doctor George E. Blackham.......... 78
Doctor J. H. Baxter................
Doctor James A Hall................ 127
Don't Grunt........................ 153
Do We Owe Our Parents Anything?. 23
Economy of Bacteria................ 193
Edison Humbug ..................... 26
Educational........................ 152
Effective Advertising.............. 47
Elmira College..................... 22
Elmira Morning Herald, The......... 255
Emphatically Settled............... 74
End of the Year.................... 204
Error in Title Page................ 22

| | Page. |
|---|---|
| Fairs and Festivals | 102 |
| Faith Cures, Geo. E. Blackham, M.D | 189 |
| Fogs | 285 |
| Fringed Gentian | 282 |
| Fritz Visits Philadelphia | 128 |
| From Camp | 126 |
| Gambling | 78 |
| Gazette, The | 126 |
| Generosity | 23 |
| Gin Mills | 228 |
| Going to Florida | 100 |
| Guiteau | 74 |
| Guiteau's Picture | 47 |
| H.'s Experiment | 47 |
| Hancock, General | 22 |
| Happenings in Camp | 154 |
| Hats for the Opera | 102 |
| Healing Art, The | 255 |
| Holidays, The | 101 |
| How to Rest | 51 |
| Hurrah | 100 |
| Ingersoll, Dr. A. J | 23 |
| "Jim." | 256 |
| Killing Mercifully | 232 |
| Kissing | 24 |
| Large Land-holder | 47 |
| Latin in Schools | 178 |
| List of New Books | 233 |
| Microscopy | 24 |
| Microscopic Germs | 179 |
| Medical Societies | 179–204 |
| Mis-named | 73 |
| Multiplication by Fission | 177 |
| National Calamity, The | 50 |
| National Board of Health | 208 |
| Nation Mourns, A | 73 |
| New Telephone | 229 |
| New Type | 22 |
| Nineteenth of the Month, The | 73 |
| North American Review, The | 73 |
| Not an Evil | 77 |
| Opera House, The | 126 |
| Oscar Wilde | 127 |
| Our Amphiuma | 256 |
| Our Contributors | 178 |
| Our Delay | 47 |
| Our Sewerage | 75 |
| Park Church Debt | 126 |
| Penalties | 47 |
| Political | 178 |
| Political Picnic, The | 100 |
| Pratt, Ben. H., in Washington | 22 |
| Premium List | 22 |
| President Garfield | 22 |
| Prison Discipline | 257 |
| Probing for Bullets | 47–73 |
| Rain | 255 |
| Rather Hard | 100 |
| Reformatory Investigation, The | 127 |
| Responsibility of the Insane | 103 |
| Revolutionary | 100 |
| Reward for Scientific Investigations | 177 |
| Sara's Art Gallery | 26 |
| Schwab | 22 |
| Seaside Resorts | 48 |
| Second Sight | 75 |
| Secretary of State | 282 |
| Sick Headache, A | 101 |
| Soiree, Microscopical | 126 |
| Specimen Copies | 23 |
| Spencer, Charles A | 101 |
| Stuffing an Owl | 101 |
| Structure of the Blood Corpuscle | 181 |
| Suicide | 283 |
| Sunday Papers | 22 |
| Sunday Tidings | 100 |
| Surgery Extraordinary | 177 |
| Surgeon General of the Army | 23 |
| Telephone Vexations | 207 |
| That Code of Medical Ethics | 232 |
| That Dorsey Letter | 227 |
| That Induction Balance | 73 |
| That New Code | 153 |
| That Sham Flopper | 231 |
| The Adirondacks | 258 |
| The Anarchists | 230 |
| The Arrangement | 22 |
| The Assassin Dukes | 232 |
| The Attending Surgeons | 76 |
| The Bacillus | 233 |
| The Contributions | 73 |
| The Dukes Case | 256 |
| The Genius of Skepticism | 128 |
| The Glorious Minority | 285 |
| The Index | 305 |
| The Office of Coroner | 207 |
| "The Old Doctor" | 231 |
| The Opera House | 282 |
| The State Fair | 77 |
| The Stratford Case | 283 |
| The Tariff | 228 |
| The Trade Dollar | 257 |
| Those Doctors' Bills | 152 |
| Two Weeks Late | 152 |
| Vaccination | 129 |
| Well Filled | 127 |
| Whistling | 77 |
| Why Defend? | 47 |
| Wiggins, Vennor | 227 |
| Woodsy | 152 |
| Worthy of Record | 78 |

mouth night and morning, and to begin the use of the tooth-brush early (likewise the tooth-pick.)

6. With regard to the food of children, to those who are old enough, whole meal bread, porridge and milk should be given. This is much more wholesome and substantial food than white bread.

7. If the foregoing instructions were carried out, comparatively few teeth would have to be extracted.

### OUR BOOK TABLE.

THE PATHOLOGY AND TREATMENT OF EPULIS. —An address delivered before the Wisconsin Dental Society at Milwaukee, by W. Senn, M. D., of Milwaukee. A paper of value to the Dentist and Surgeon.

THE MEDICAL SUMMARY.—A monthly journal of Practical Medicine, New Preparations, etc., by R. H. Andrews, M. D., Lansdale, Pa. Terms, $1 per year. A useful little magazine and very cheap at the price asked.

OPTICO-CILIARY NEUROTOMY, a pamphlet by Julian J. Chisolm, M. D., Professor of Eye and Ear Surgery in University of Maryland, etc. A treatise on a proposed substitute for extirpation of a lost and painful eye ball. To be had of the author, Baltimore, Md.

THE PITTSBURG MEDICAL JOURNAL, a monthly journal devoted to Medicine and Surgery, Robert C. Gallaher, M. D., Editor and Proprietor, $2 per year. A new arrival in the field of medical literature. A long list of able contributors appears upon the title page.

THE DEVELOPMENT OF THE OSSEOUS CALLUS, in fractures of the bones of Man and Animals, by Henry O. Marcy, A. M., M. D., Cambridge, Mass. The results of investigations upon the reparative processes in fractures, in which some new and novel ideas are expressed.

THE CINCINNATI LANCET AND CLINIC, a weekly journal of Medicine and Surgery, issued every Saturday by J. C. Culbertson, M. D., and J. G. Hyndman, M. D., at $3.00 per annum. Address 281 W. 7th St., Cincinnati, O. A sprightly, well-edited journal, with an able corps of contributors.

THE CHICAGO MEDICAL REVIEW, published on the fifth and twentieth of every month at $2 per year, at 60 Wabash Av., Chicago, Ills. The best edited medical journal that comes to our table, and one of the very best adapted to the wants of the busy practitioner of medicine, who cannot spare the time to read a weekly.

PHTHISIS PULMONALIS, by L. de Bremon, M. D., University of Paris, France. Reprint by John Nerston, 36 Beekman St., New York.—A pamphlet on the treatment of this disease with the Hypophosphites. A scientific paper that should be read by every physician interested in the treatment of this distressing disease. Price of pamphlet not stated.

HAND-BOOK OF URINAY ANALYSIS; Chemical and Microscopical, by Frank M. Deems, M. D., Laboratory Instructor in the Medical Department of the Univ. of N. Y.—Industrial Publication Co. A very valuable hand book that will be found to be of practical utility to every physician. The very insignificant price, 25 cents, makes it readily obtainable.

THE INTERNATIONAL JOURNAL OF MEDICINE AND SURGERY, published weekly, Publication office No. 1 Chambers St., New York. Price $5 per annum. This is a new journal that means to occupy a field not hitherto attended to, to-wit: The complete and faithful translations of the best medical literature of the various foreign journals. The initial numbers present much valuable matter.

THE NORTH AMERICAN REVIEW, edited by Allen Thorndike Rice.—Published by D. Appleton & Co., New York, at $5 per year. To use a popular term, this is the "stalwart" of American magazines. The contributions are from the best known American writers, and upon living, and therefore interesting themes. No one who has a desire for the best literature can afford to be without this magnificent magazine.

HOW TO SEE WITH THE MICROSCOPE,—is the title of a neatly-printed and illustrated book of over 400 pages, upon the subject which its title fully indicates. It is by J. Edwards Smith, of Cleveland, O., and is published by Duncan Bros., Chicago. Price, $2.00. Perhaps there is no microscopist more capable of giving genuine instruction in the management of the microscope, than the author referred to. We are sorry that the valuable information contained in this book should be interspersed with so much slang,—it mars the otherwise really valuable text.

### THE BISTOURY:

Thad S. Up de Graff, M. D., Editor & Proprietor.

### *CONTENTS FOR APRIL, 1881.*

**CONTRIBUTIONS.** PAGE.

Capital Impressions .......................... BEN. H. PRATT, .......................................... 1

The Book Wonderful, .......................... AUSBURN TOWNER .......................................... 4

Protoplasm .......................... S. O. GLEASON, M. D .......................................... 6

Words and Slang .......................... J. B. CHANDLER .......................................... 7

To Prevent the Spreading of Scarlet Fever . . J. DAVIDSON, M. D. .......................................... 8

**TERMS.—50 Cents a Year. Address The Bistoury, Elmira, N. Y.**

---

### SUBSCRIPTIONS RECEIVED TO APRIL, 1881.

NEW YORK—A Tillotson, N Graves, W O Osborne, Dr H M Bolles, Minnie B DeLand, I D Hopkins, Dr R N Cooley, E Robinson, Dr J Angel $2, Jno Callister, E D Mills, Miss T Miller, A P Merchant $1, H R Rogers, J T Nichols, D A Chatfield $1, C T Bush, Robt T Turner, Dr J M Hagey, L H Ainsworth, H B Gibert, H M Paine, A C McFarren, Thos M Howell, A Houghton Jr, Dr S G Ellis, Jesse Owen, Dr H D Wells, Theo L Minier, Ingraham Bros, L A Humphrey, Dr F B Darby, F G Hall, S S Hamlin, Thos Johnson, Dr E D Leonard, Stuart & Beach, Dr L D Parkhurst, Bundy Bros, N P Fassett, H B Berry, S Dewitt, M S Palmer, W F Wentz, G P Rawson, Dr S M Eddy $1, J Secor, E Goddard, Mrs Chas Rapelyea, Chas Langdon, W M Sanders, J H Cottrell $1, W J Lormore, E B Youmans, Thos Lormore, C M Beadle, E G Baldwin, M S Converse, Mrs C Preswick.

PENNSYLVANIA—H Ross $5, J W Stevens, E Covell $2 50, Dr S W Dayton, Dr D J Hillbish, Dr J Davidson $1, J H McMinn, Dr S S Simmons, A A Root $1, J F Kocher, Alex Beade, Jno Y Shindel $1, Dr S B Hartman $1, Jas B Chandler, W C Richardson, Dr T J Birch, Rev R Smith, R S Wallace, K A Lowell, W H Rowig, B L Anton, G M Rowig, A Harshberger $2, Dr R Myers $2, Robt Thomas, J R Shellenberger, Prof A S Low, Mrs E A Smead, Wm Stewart.

ALABAMA—Dr J W Burton, L O Zeigler, J C Yarbrough, Dr C Crosman, Dr D D Kertzog.

ARKANSAS—Dr S R Mason, Dr L H Hall, Dr A Maddill, Dr Flemming.

CALIFORNIA—Clarke Lewis, Dr J F Gibbon, Dr. Jas B Cook, Dr Felix Strom.

COLORADO—Dr H Sommers, Dr L N Beech, Drs Hamlin & March.

CONNECTICUT—Dr G H Miner, Dr Gallager, Dr H A Sampson, Dr N D Cook, Dr F A Folks, Dr H Stephenson.

DELAWARE—Dr Homer Scott, Dr N A Harris, Dr O N Smith.

DISTRICT OF COLUMBIA—N N Blunt, Dr Harvey Smith.

FLORIDA—Dr A Manifieler, E J Harris, Dr E T Sabal, Calvin Oak, Dr N C Stevens.

GEORGIA—Dr M Meichselbaum, Dr S H Gray, Dr B F Ruddisill.

ILLINOIS—Dr W H Crane $1, Dr J A Hubbard $2, Dr S Stephenson, Thos L Carey.

INDIANA—L O'Neal $1, Dr O de Kress.

IOWA—Dr J Butts.

KENTUCKY—Dr Thos Lyon $3, Dr B B Harris $5.

LOUISIANA—Dr S R Louuberger $2, Dr P N Pennebaker $1.

MAINE—O N Snow, Dr B Ramsdell.

MARYLAND—Dr Z R Morgan, Dr Jerome Lyons $2.

MASSACHUSETTS—Miss Howland, Dr D B Sayles $1, Dr Sam Houston.

MICHIGAN—W S Livermore, S Dubois, Dr L W Farquelle, Mrs C J Davis, Dr J W Babbitt $1, Dr H Loomis $1.

MINNESOTA—J W Foss, R P Wells, J T Cook, Mrs A W Wells.

MISSISSIPPI—Dr P Lamont $2, Dr Jas Houston $1.

MISSOURI—M Wahl, Miss F Baier, G E Buxton $1.

NEBRASKA—Mrs Jas Harrison, Dr H Hamilton $2.

NEVADA—Dr Dan Blampied, Dr D H Blossom.

NEW HAMPSHIRE—Dr S M Emery, Dr Hildebrand $2,

NEW JERSEY—S E Burr 1$, H H Bradley, Dr R R Hoose $2.

NORTH CAROLINA—Dr F L James.

OHIO—Dr D Kitchen, F R Holley $3.

SOUTH CAROLINA—D L Anderson, Dr P Smith.

TENNESSEE—Dr J P Dake, Dr L Habberstraw.

TEXAS—Dr Snodgrass, Dr Fitzpatrick.

VERMONT—C F Barrett.

WEST VIRGINIA—Dr Erenberg.

WISC ONSIN—G W Sheardown, P N Shores.

WASH INGT ON TERRITORY—Jas Alexander, Mrs P Flourey $2.

